# Pre-treatment peripheral absolute monocyte count predicts metastatic progression and survival outcomes in treatment-naive non-metastatic nasopharyngeal carcinoma

**DOI:** 10.3389/fonc.2026.1696050

**Published:** 2026-02-17

**Authors:** Fen Cai, Shuopo Fang, Jianfeng Cai, Guodong Qiu, Xiaorui Cai

**Affiliations:** 1 Department of Nosocomial Infection Management, Cancer Hospital of Shantou University Medical College, Shantou, Guangdong, China; 2 Department of Pharmacy, Cancer Hospital of Shantou University Medical College, Shantou, Guangdong, China; 3 Department of Interventional Therapy, Cancer Hospital of Shantou University Medical College, Shantou, Guangdong, China

**Keywords:** absolute monocyte count, metastasis, nasopharyngeal carcinoma, overall survival, prognostic biomarker, treatment-naive

## Abstract

**Background:**

The tumor node metastasis (TNM) staging system does not fully capture tumor heterogeneity, underscoring the needs for reliable biomarkers for prognostication in non-metastatic nasopharyngeal carcinoma (NPC). While inflammatory markers have shown potential, the prognostic value of pre-treatment peripheral absolute monocyte count (AMC) in treatment-naive, non-metastatic NPC remains underexplored.

**Methods:**

This retrospective cohort study analyzed 2,046 patients with newly diagnosed treatment-naive, non-metastatic NPC from 2009 to 2022. The optimal AMC cut-off value (0.63×10^9^/L) for distant metastasis-free survival (DMFS) was determined using maximally selected rank statistics method. Patients were stratified into low AMC (<0.63×10^9^/L, n=1370) and high AMC (≥0.63×10^9^/L, n=676) groups. The primary endpoints were DMFS, bone metastasis-free survival (BMFS), and overall survival (OS). Associations were assessed using Kaplan–Meier analysis, log-rank tests, Cox proportional hazards models, restricted cubic splines (RCS), subgroup analyses and sensitivity analyses. Diagnostic performance was evaluated using time-dependent receiver operating characteristic (tROC) analysis at 1, 3, and 5 years.

**Results:**

Over a median follow-up of 77.4 months, distant metastasis, bone metastasis, and death occurred in 13.5%, 6.9%, and 24.2% of patients, respectively. High pre-treatment AMC was independently associated with significantly worse DMFS (hazard ratio [HR]=1.33, 95% confidence interval [CI]: 1.05–1.70, P = 0.020), BMFS (HR = 1.86, 95%CI: 1.33–2.60, P<0.001), and OS (HR = 1.34, 95%CI: 1.11–1.61, P = 0.002) in fully adjusted models. RCS revealed a linear association between AMC and metastasis risk (P for non-linearity >0.05), but a non-linear threshold effect for OS (P for non-linearity=0.011). The risk of all-cause mortality increased with AMC >0.54×10^9^/L, eventually reaching a plateau. Subgroup analyses confirmed the feasibility of AMC’s prognostic value across all patient strata (P for interaction>0.05). Time-dependent ROC analysis demonstrated the highest discriminatory accuracy for predicting 1-year bone metastasis (AUC = 0.720), while maintaining moderate prognostic utility for distant metastasis and overall survival across 1 to 5 years.

**Conclusion:**

Pre-treatment peripheral AMC ≥0.63×10^9^/L could serve as an independent predictor of metastatic progression, particularly bone metastasis, and reduced survival in treatment-naive, non-metastatic NPC. Being easily obtainable via routine complete blood count, AMC provides significant prognostic value to enhance clinical risk stratification and guide individualized treatment planning.

## 1 Introduction

Nasopharyngeal carcinoma (NPC) is a malignant tumor that originates from the nasopharyngeal epithelium and is closely associated with Epstein–Barr virus (EBV) infection, characterized by low differentiation and high malignancy (1, 2). According to the International Agency for Research on Cancer, the number of new cases and new deaths from NPC in 2022 were 120,416 (ranking 23rd) and 73,476 (ranking 21st), respectively, accounting for only 0.6% of all diagnosed cancer cases and 0.8% of all cancer-related deaths (3). Although the incidence and mortality rates of NPC are relatively low compared to those of other cancer types, NPC exhibits remarkable racial and geographic distribution. Nearly half of the global NPC cases occur in China, particularly among the Cantonese population in Guangdong, which exhibit the highest incidence rate (4).

Early diagnosis of NPC is challenging and 60–70% of patients with the disease are diagnosed at an advanced stage (Stages III and IV) (5). Due to its deep-seated anatomical location and radiosensitive characteristics, radiotherapy with or without chemotherapy is regarded as the standard treatment for NPC, while surgical resection is infrequently necessary (6). The combination of current standardized radiotherapy and chemotherapy protocols has substantially increased the survival rates for patients with NPC (7). Furthermore, targeted therapy and immunotherapy have improved treatment outcomes for some patients with NPC (8). Reports indicated that the estimated 5-year overall survival (OS) rates after radiotherapy for Stages I and II NPC are 98% and 92%, with local failure-free survival (FFS) rates of 98% and 94%, and distant FFS rates of 98% and 91%, respectively (9). Although the use of combined chemoradiotherapy as the primary treatment strategy has improved the overall prognosis for patients with locally advanced NPC, 20–30% of patients still experience local recurrence and distant metastasis, resulting in a median survival of less than 2.5 years (10, 11). Primary tumor cells of NPC initially migrate to regional lymph nodes and subsequently disseminate to bones, inguinal lymph nodes, or the liver through distinct routes (12). A study conducted in South India reported that lymph node metastasis was present in 90% of patients and distant metastasis was present in 16% of patients, predominantly affecting the bones, lungs, and liver (13). Additionally, a study in Indonesia indicated that over 30% of patients with NPC were already at advanced stages upon consultation, with bone metastasis being most prevalent, which significantly affected their OS (14). Moreover, the 5-year survival rate for patients with recurrent NPC after re-radiotherapy was reported to be only 27.5–57.2% (15). Retrospective studies have demonstrated significant variations in survival outcomes based on the anatomical sites involved and the number of metastatic lesions (16, 17). For specific subgroups, the OS may exceed 10 years (17). However, due to the heterogeneity of NPC, patients with the same tumor node metastasis (TNM) staging may experience significantly different prognosis, even after receiving similar treatment modalities (18, 19). These findings indicate that TNM staging is insufficient for predicting prognosis and fail to adequately reflect tumor biological heterogeneity. The limitations of the traditional TNM staging system in predicting prognosis for NPC are becoming increasingly evident (20). Therefore, identifying effective and accurate prognostic indicators is of great significance for risk stratification and individualized treatment of NPC.

Inflammation is a critical component of the tumor microenvironment (TME) and is closely related to tumor occurrence, development, and prognosis (21, 22). It can influence tumor invasion and metastasis through DNA damage, inhibition of apoptosis, and promotion of angiogenesis (23). Several studies have suggested that inflammation-related indexes derived from routine complete blood tests, including neutrophils, lymphocytes, platelets, monocytes, neutrophil to lymphocyte ratio (NLR), platelet-to-lymphocyte ratio (PLR), and lymphocyte to monocyte ratio (LMR), may serve as predictive factors for the diagnosis and prognosis of various tumors and non-neoplastic diseases, as well as provide insights into a patient’s immune status (24, 25). Elevated pre-treatment peripheral lymphocyte count is associated with favorable prognosis in Hodgkin’s lymphoma (26), non-small cell lung cancer (27), and metastatic gastric cancer (28). Conversely, a high monocyte level has been identified as a negative independent prognostic indicator for individuals diagnosed with diffuse large B-cell lymphoma and metastatic melanoma (29). Previous studies have shown that NLR, LMR, and PLR are closely associated with the prognosis of osteosarcoma (30), cholangiocarcinoma (31), and rectal cancer (32). Similarly, corresponding studies have been reported on NPC, but the results seem conflicting (33–35). Therefore, some researchers have expressed concerns regarding the reproducibility and reliability of inflammatory cell-based scoring in clinical practice. For example, the variability in prognostic cut-off values for NLR, PLR, and LMR, as well as the non-tumor-specific nature of these indicators, suggests their relationship with NPC prognosis requires further confirmation (36).

Monocytes can differentiate into macrophages and myeloid dendritic cells, serving as key immune cells in inflammatory responses (37). Recent studies have demonstrated that monocytes infiltrate various tumors and are frequently found in the TME. They modulate the TME by inducing increased chemotherapy and immune tolerance, metastasis and diffusion, angiogenesis, and tumor cell dissemination (38). The impact of monocytes on prognosis varies among different tumor types. Research has indicated that a low preoperative absolute monocyte count (AMC) can predict OS benefits in patients with oral squamous cell carcinoma (39). In patients with castration-resistant prostate cancer undergoing docetaxel chemotherapy, elevated monocyte counts correlate with poorer survival outcomes (40). Among patients with esophageal squamous cell carcinoma undergoing esophagectomy, a higher preoperative AMC is independently associated with lower disease-free survival (DFS) and OS (41). Additionally, monocyte count has been independently correlated with the prognosis in malignant tumors such as gastric cancer, acute lymphoblastic leukemia, lymphoma, and hepatocellular carcinoma (42). To the best of our knowledge, a comprehensive analysis of AMC in patients with newly diagnosed treatment-naive, non-metastatic NPC remains limited. Therefore, we conducted this retrospective cohort analysis to determine the prognostic value of pre-treatment peripheral AMC in predicting distant metastasis, bone metastasis, and all-cause mortality in NPC, establishing a foundation for clinical decision-making regarding patient risk stratification and individualized treatment strategies.

## 2 Methods

### 2.1 Patients

**Figure 1:**
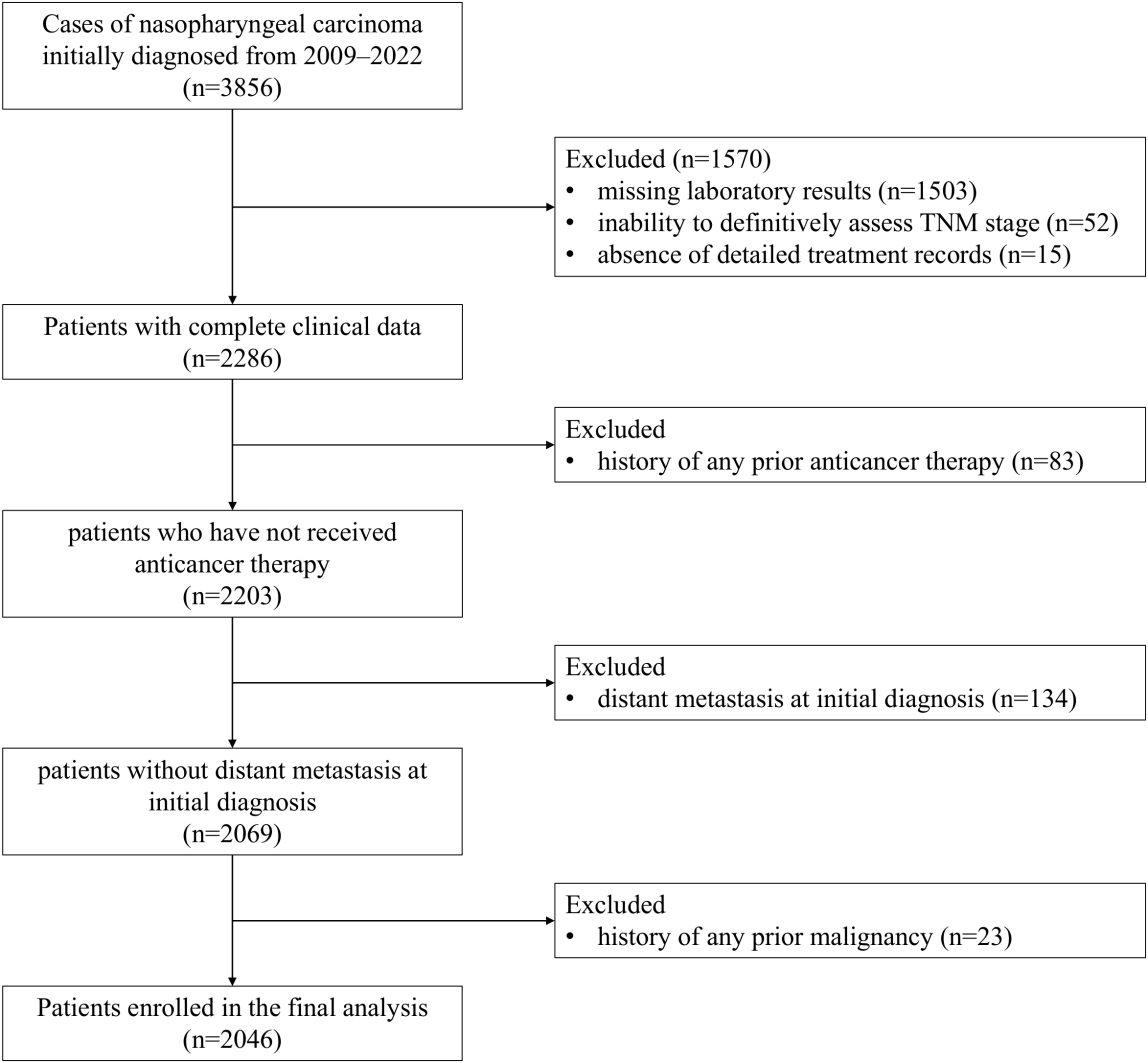
The flowchart of patient selection.

In this retrospective study, we analyzed 3,856 patients newly diagnosed with NPC at the Cancer Hospital of Shantou University Medical College between January 2009 and January 2022. Patients were excluded based on the following criteria: (1) incomplete clinical data, including missing laboratory results, inability to definitively assess TNM stage, or absence of detailed treatment records; (2) a history of any prior anti-cancer therapy, including chemotherapy, radiotherapy, surgery or other therapy for NPC; (3) presence of distant metastasis at the time of initial diagnosis; (4) history of any prior malignancy. The patient selection process is detailed in **Figure 1**. After applying the exclusion criteria, a final cohort of 2,046 eligible patients were included in the subsequent analyses. To address potential selection bias arising from missing laboratory data, which accounted for the exclusion of 1,503 patients, we compared key baseline characteristics between included and excluded individuals. As presented in **Supplementary Table S1**, there were no statistically significant differences in age (P = 0.333), sex distribution (P = 0.350), or TNM stage (P = 1.000) between the two groups. This study was conducted in accordance with the principles of the Declaration of Helsinki and received approval from the Ethics Committee of the Cancer Hospital of Shantou University Medical College (Approval No.: 2022039). The requirement for written informed consent was waived by the approving Ethics Committee owing to the retrospective nature of the study design.

### 2.2 Data collection and follow-up

Baseline clinical and laboratory parameters were extracted from the Hospital Information System (HIS) using the first recorded hospitalization data prior to the initiation of any anti-cancer treatment. Data collected included demographics (age and sex), TNM stage classified according to the contemporary American Joint Committee on Cancer/Union for International Cancer Control TNM staging system at diagnosis, and treatment modalities (radiotherapy, chemotherapy, concurrent chemoradiotherapy) at initial hospital admission. Treatment parameters such as dosage, regimen, and administration methods were not considered in this analysis. Comprehensive laboratory parameters encompassed complete blood count components, specifically absolute monocyte count (AMC), white blood cell count (WBC), absolute neutrophil count (ANC), absolute lymphocyte count (ALC), red blood cell count (RBC), hemoglobin (HGB), and platelet count (PLT). Hepatic and renal function was assessed based on aspartate aminotransferase (AST), alanine aminotransferase (ALT), alkaline phosphatase (ALP), total bilirubin (TBIL), blood urea nitrogen (BUN), and creatine kinase (CK) measurements. Serological status for hepatitis B surface antigen (HBsAg) and anti-hepatitis C virus (anti-HCV) were also recorded.

Patients were prospectively followed up via scheduled clinical visits. For those without scheduled inpatient or outpatient visits, annual telephone follow-ups with patients or designated relatives were conducted to assess survival outcomes. Comprehensive survival surveillance was maintained until February 7, 2025. The primary endpoint was distant metastasis-free survival (DMFS), defined as the duration from initial hospital admission until the first confirmed diagnosis of distant metastasis through radiological or histopathological verification. Patients who remained free of distant metastasis were censored at the date of their most recent hospitalization, at which point their distant metastasis-free status was confirmed. The secondary endpoints included bone metastasis-free survival (BMFS), measured from initial hospital admission until the initial detection of bone metastasis confirmed through standard imaging techniques including bone scan, PET-CT, or MRI. Similarly, the censoring time point for patients without bone metastasis was defined as the date of their last documented hospital admission, when the absence of bone metastasis was confirmed. Additionally, OS was assessed from the admission date until death from any cause, with surviving patients censored at their last confirmed contact date.

### 2.3 Statistical analysis

Continuous variables, assessed for normality using the Shapiro–Wilk test, were reported as median (interquartile range, IQR) and compared using Mann–Whitney U tests. Categorical variables were expressed as frequencies (%) and analyzed using Pearson’s chi-squared or Fisher’s exact tests. The optimal cut-off value for AMC corresponding to the most significant association with distant metastasis outcomes was determined with the R package “maxstat” (Maximally Selected Rank Statistics), which were then used to categorize participants into high and low AMC groups. Survival curves were generated using the Kaplan–Meier method and compared using log-rank tests. Cox proportional hazards models were constructed to evaluate the association between AMC and distant metastasis as well as bone metastasis and all-cause mortality. The results were expressed as hazard ratios (HRs) and 95% confidence intervals (CIs). Three models were constructed to adjust for possible confounding factors. Model 1 was adjusted for no variable; Model 2 incorporated age, sex, and TNM stage; and Model 3 further adjusted Model 2 to account for additional factors including radiotherapy and chemotherapy. The “rms” package was used to plot restricted cubic splines (RCS) with four knots to visualize the potentially non-linear association between AMC and distant metastasis as well as bone metastasis and all-cause mortality. To evaluate whether any significant interaction exists between these factors and AMC when analyzing distant metastasis, bone metastasis and all-cause mortality, we conducted subgroup analyses stratified by age, sex, radiotherapy, chemotherapy, and concurrent chemoradiotherapy. To assess the robustness of the primary findings against potential confounding, sensitivity analyses were performed and the primary multivariable Cox proportional hazards models (Model 3) were refitted in each resultant subset. To dynamically assess the prognostic accuracy of AMC over time, we performed time-dependent receiver operating characteristic (tROC) analysis and calculated the area under the curve (AUC) with 95%CI at 1, 3, and 5 years for distant metastasis, bone metastasis and all-cause mortality. All statistical analyses were performed with R software (Version 4.4.3). A two-tailed P-value of <0.05 was considered to indicate statistical significance.

## 3 Results

### 3.1 Demographic and clinical characteristics of the study population

A total of 2,046 patients with NPC were enrolled and stratified into the low AMC group (n=1,370; AMC <0.63×10^9^/L) and high AMC group (n=676; AMC ≥0.63×10^9^/L) using the optimal cut-off value identified using the maxstat package. Age distribution was comparable between the two groups. Notably, the high AMC group included a higher proportion of male patients relative to the low AMC group. TNM stage distribution differed, with the high AMC group containing a higher proportion of advanced-stage (Stage IV) cases. Treatment patterns, including radiotherapy, chemotherapy, and concurrent chemoradiotherapy, were similarly distributed across both groups. Hematological parameters (WBC, ANC, ALC, RBC, PLT, and HGB levels) were generally elevated in the high AMC group. Additionally, biochemical marker levels (AST, ALT, and ALP) were higher in the high AMC group than in the low AMC group. Infection statuses, assessed via anti-HCV positivity and HBsAg positivity, were comparable between the low and high AMC groups. Additional baseline characteristics of the participants are presented in **Table 1**.

**Table 1:** General characteristics for included patients between low and high AMC groups.

Variables	Low-AMC (n=1370)	High-AMC (n=676)	*P* value
Demographic characteristics			
Age (years)	51.00 (44.00,59.00)	51.00 (44.00,58.00)	0.626
Male, n (%)	925.00 (67.50)	590.00 (87.30)	<0.001
Tumor status			
TNM stage, n (%)			<0.001
I	31.00 (2.30)	6.00 (0.90)	
II	132.00 (9.60)	45.00 (6.70)	
III	684.00 (49.90)	295.00 (43.60)	
IV	523.00 (38.20)	330.00 (48.80)	
Treatments			
Radiotherapy, n (%)	1154.00 (84.20)	562.00 (83.10)	0.568
Chemotherapy, n (%)	1214.00 (88.60)	610.00 (90.20)	0.301
Concurrent chemoradiotherapy, n (%)	1004.00 (73.30)	497.00 (73.50)	0.952
Hematological parameters			
WBC, ×10^9^/L	6.39 (5.40,7.30)	8.27 (7.24,9.76)	<0.001
ANC, ×10^9^/L	3.80 (3.08,4.63)	5.03 (4.10,6.30)	<0.001
ALC, ×10^9^/L	1.80 (1.44,2.20)	2.10 (1.69,2.60)	<0.001
RBC, ×10¹²/L	4.70 (4.35,5.01)	4.75 (4.41,5.08)	0.003
PLT, ×10^9^/L	232.80 (199.00,273.82)	252.00 (211.65,310.00)	<0.001
HGB, g/L	137.45 (127.80,146.57)	141.00 (131.57,149.10)	<0.001
Biochemical markers			
AST, U/L	20.60 (17.00,25.00)	21.00 (17.00,26.42)	0.015
ALT, U/L	22.00 (17.00,29.00)	24.00 (18.78,33.00)	<0.001
ALP, U/L	87.30 (70.00,107.00)	92.00 (75.00,115.00)	<0.001
TBIL, μmol/L	14.60 (12.80,17.30)	14.40 (12.47,17.00)	0.139
BUN, mmol/L	4.69 (3.91,5.64)	4.81 (3.89,5.81)	0.228
CK, U/L	87.00 (66.60,118.07)	89.00 (66.00,118.85)	0.910
Immunological examination			
Anti-HCV positive, n (%)	5.00 (0.40)	6.00 (0.90)	0.195
HBsAg positive, n (%)	189.00 (13.80)	115.00 (17.00)	0.063

### 3.2 Survival analysis of AMC in patients with NPC

**Figure 2:**
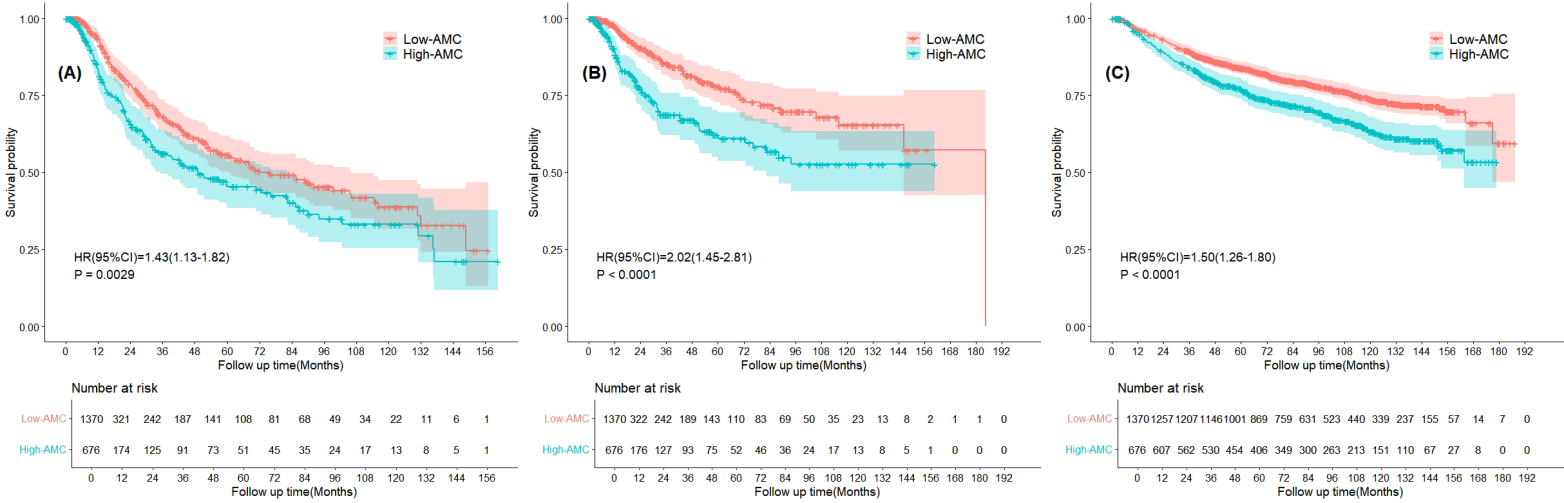
Kaplan–Meier curves of the survival rates of NPC participants with low (<0.63×10^9^/L) and high (≥0.63×10^9^/L) AMC. **(A)** distant metastasis-free survival. **(B)** bone metastasis-free survival. **(C)** overall survival.

During a median follow-up of 77.4 months (IQR 43.0–118.6 months), distant metastasis developed in 276 patients (13.5%), bone metastasis occurred in 142 individuals (6.9%), and 495 cases of deaths (24.2%) were documented. To evaluate the prognostic impact of AMC, we performed Kaplan–Meier survival analysis with log-rank tests to compare survival outcomes between patients stratified by the optimal AMC cut-off value (0.63×10^9^/L) derived from the maxstat package (**Figure 2**). Three key survival endpoints —DMFS, BMFS, and OS—were assessed. The Kaplan–Meier curves for DMFS revealed a significant divergence in cumulative survival probabilities between the low and high AMC groups (log-rank test, P = 0.0029). Throughout the follow-up period, the high AMC group consistently exhibited lower DMFS probabilities compared to the low AMC group (**Figure 2A**). For BMFS, the log-rank test confirmed a marked survival disparity between the two groups (P<0.001). The high AMC group demonstrated a more pronounced reduction in cumulative BMFS probabilities, with survival curves separating earlier and maintaining a wider gap over time (**Figure 2B**). In the OS analysis, the Kaplan–Meier curves showed a clear separation in cumulative survival rates, with the high AMC group experiencing significantly poorer OS than the low AMC group (log-rank test, P<0.001). Survival differences were evident from the early follow-up period, with the low-AMC group maintaining higher survival probabilities across all time points (**Figure 2C**).

### 3.3 Association between AMC and survival outcomes

To evaluate the prognostic significance of AMC, we performed Cox proportional hazards regression analysis across three key survival endpoints—distant metastasis, bone metastasis, and all-cause mortality (**Table 2**). Three models were constructed—Model 1 (unadjusted), Model 2 (adjusted for age, sex, and TNM stage), and Model 3 (adjusted for age, sex, TNM stage, radiotherapy, and chemotherapy). For distant metastasis, high AMC (≥0.63×10^9^/L) was associated with an increased risk across all models. Model 1 yielded an HR of 1.43 (95%CI: 1.13–1.82, P = 0.003); Model 2 showed a slightly attenuated HR of 1.30 (95%CI: 1.02–1.65, P = 0.033); and Model 3 maintained significance with an HR of 1.33 (95%CI: 1.05–1.70, P = 0.020). Notably, the association between elevated AMC and bone metastasis demonstrated the greatest magnitude across all endpoints. Model 1 revealed a 2.02-fold increased risk (HR = 2.02, 95%CI: 1.45–2.81, P<0.001); Model 2 showed a reduced risk to 1.82 (95%CI: 1.30–2.54, P<0.001); and Model 3 retained significance with an HR of 1.86 (95%CI: 1.33–2.60, P<0.001). Additionally, high AMC was predictive of all-cause mortality. Model 1 showed an HR of 1.50 (95%CI: 1.26–1.80, P<0.001); Model 2 yielded an HR of 1.36 (95%CI: 1.13–1.64, P = 0.001); and Model 3 maintained a significant association with an HR of 1.34 (95%CI: 1.11–1.61, P = 0.002).

**Table 2:** Cox proportional hazards regression analysis of AMC and outcomes in NPC patients.

Outcomes	Model 1			Model 2			Model 3		
	HR	95%CI	*P* value	HR	95%CI	*P* value	HR	95%CI	*P* value
Distant metastasis									
Low-AMC	1.00			1.00			1.00		
High-AMC	1.43	1.13-1.82	0.003	1.30	1.02-1.65	0.033	1.33	1.05-1.70	0.020
Bone metastasis									
Low-AMC	1.00			1.00			1.00		
High-AMC	2.02	1.45-2.81	<0.001	1.82	1.30-2.54	<0.001	1.86	1.33-2.60	<0.001
All-cause mortality									
Low-AMC	1.00			1.00			1.00		
High-AMC	1.50	1.26-1.80	<0.001	1.36	1.13-1.64	0.001	1.34	1.11-1.61	0.002

Model 1 adjusted for no variables.

Model 2 adjusted for age, sex, TNM stage.

Model 3 adjusted for age, sex, TNM stage, radiotherapy, chemotherapy.

### 3.4 Non-linear associations between AMC and survival outcomes

**Figure 3:**
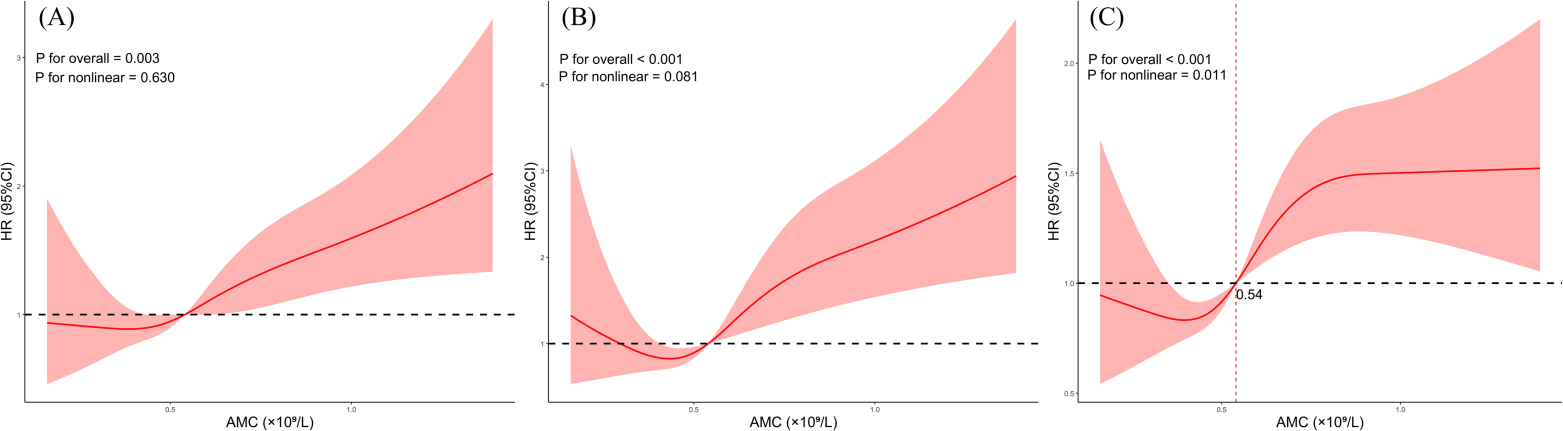
The association of AMC with distant metastasis **(A)**, bone metastasis **(B)** and all-cause mortality **(C)** among NPC participants visualized by restricted cubic spline.

The RCS analysis demonstrated a significant nonlinear relationship between AMC and the risk of all-cause mortality among patients with NPC (P for non-linearity=0.011). The AMC had an HR of 1 at the optimal cut-off value of 0.54×10^9^/L. The risk of all-cause mortality among patients with NPC increased with an increased AMC, but the rate of this increase diminished over time, eventually reaching a plateau (**Figure 3C**). However, in contrast to the relationship with all-cause mortality, the RCS analysis showed no significant non-linear relationship between AMC and the risk of distant metastasis (P for non-linearity=0.630) (**Figure 3A**). Similarly, the RCS analysis failed to demonstrate a significant nonlinear relationship between AMC and the risk of bone metastasis (P for non-linearity =0.081) (**Figure 3B**).

### 3.5 Subgroup analyses

**Figure 4:**
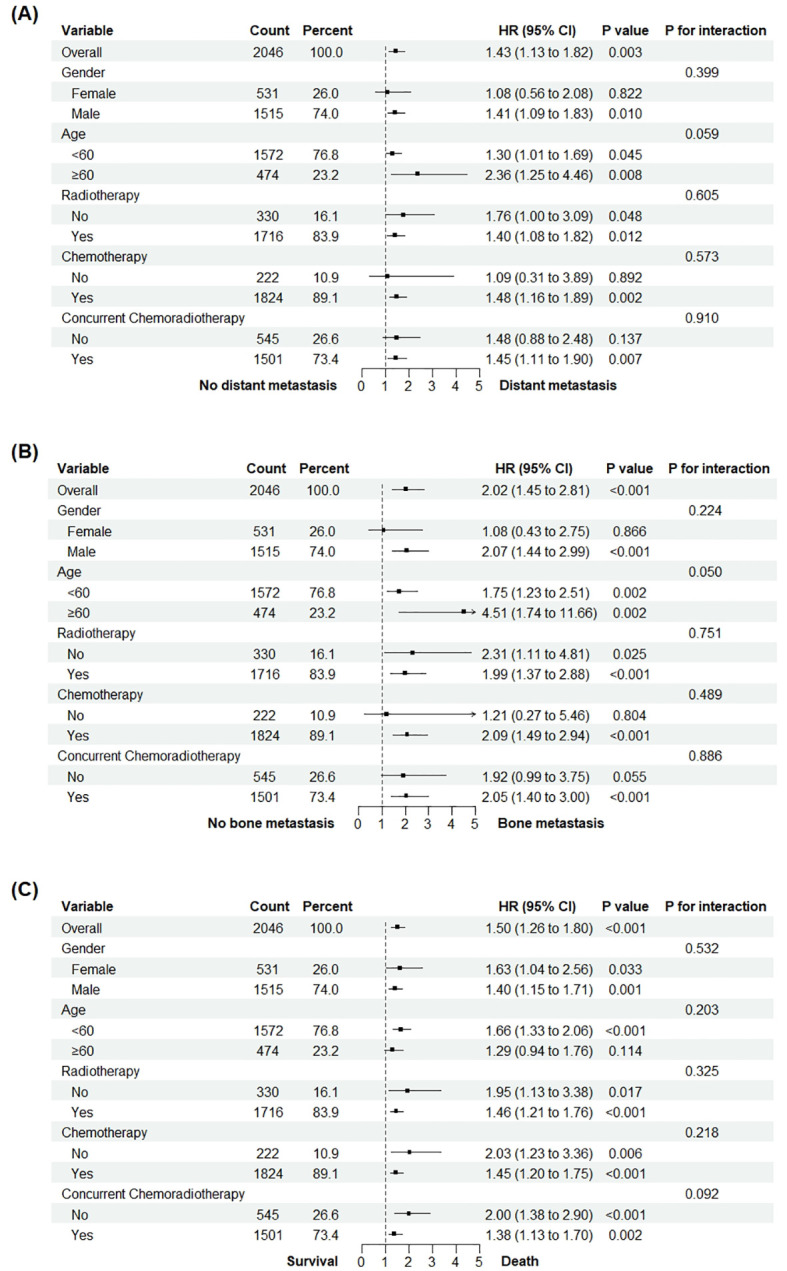
Subgroup analysis of the associations between AMC and distant metastasis **(A)**, bone metastasis **(B)** and all-cause mortality **(C)** among NPC participants.

To assess the consistency of the associations between AMC and distant metastasis, bone metastasis, and all-cause mortality across diverse subgroups, subgroup and interaction analyses were conducted, as presented in the forest plot (**Figure 4**). The findings revealed a significant association between AMC and distant metastasis in patients with NPC (HR = 1.43, 95%CI: 1.13–1.82, P = 0.003). Crucially, the P-value for interaction among subgroups exceeded 0.05, signifying that the impact of AMC on distant metastasis was stable regardless of variations in patient characteristics, including sex, age, radiotherapy status, chemotherapy status, and concurrent chemoradiotherapy status (**Figure 4A**). For bone metastasis, AMC exhibited a notable association in patients with NPC (HR = 2.02, 95%CI: 1.45–2.81, P<0.001). Moreover, the absence of a significant interaction (P for interaction>0.05) suggested that the effect of AMC on bone metastasis remained uniform across different patient subgroups (**Figure 4B**). The AMC was found to be associated with all-cause mortality in patients with NPC (HR = 1.50, 95%CI: 1.26–1.80, P<0.001). The lack of significant interactions among subgroups (P for interaction>0.05) indicated that the influence of AMC on all-cause mortality was consistent across different patient features (**Figure 4C**).

### 3.6 Sensitivity analyses

The independent prognostic value of high pre-treatment AMC (≥ 0.63×10^9^/L) remained consistent in sensitivity analyses designed to address potential confounding by inflammatory conditions (**Supplementary Table S2**). First, after excluding patients with AMC value in the extreme tails of the distribution (≤0.5th percentile [value×10^9^/L] and ≥99.5th percentile [value×10^9^/L], n=22), high AMC remained significantly associated with worse DMFS (HR = 1.31, 95%CI: 1.03–1.67, P = 0.029), BMFS (HR = 1.83, 95%CI: 1.30–2.57, P<0.001), and OS (HR = 1.34, 95%CI: 1.12–1.62, P = 0.002) in fully adjusted models. Second, after excluding patients seropositive for HBsAg or anti-HCV (n=314), the associations persisted for DMFS (HR = 1.39, 95%CI: 1.06–1.82, P = 0.017), BMFS (HR = 2.00, 95%CI: 1.38–2.92, P<0.001), and OS (HR = 1.36, 95%CI: 1.11–1.67, P = 0.003).

### 3.7 Time-dependent ROC curve analysis

**Figure 5:**
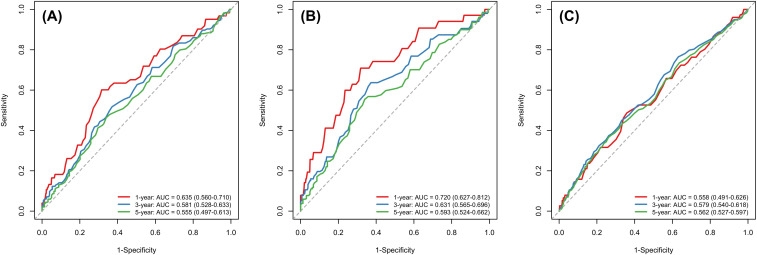
Time-dependent ROC curves of AMC for predicting distant metastasis **(A)**, bone metastasis **(B)** and all-cause mortality **(C)** among NPC participants.

We evaluated the dynamic discriminative ability of pre-treatment AMC using time-dependent ROC analysis at 1, 3, and 5 years (**Figure 5**). For predicting bone metastasis, AMC demonstrated good early performance with a 1-year AUC of 0.720 (95%CI: 0.627–0.812). The predictive accuracy remained notable at 3 years (AUC = 0.631, 95%CI: 0.565–0.696) and 5 years (AUC = 0.593, 95%CI: 0.524–0.662). For distant metastasis, the 1-year AUC was 0.635 (95%CI: 0.560–0.710), declining moderately to 0.581 (0.528–0.633) at 3 years and 0.555 (0.497–0.613) at 5 years. In predicting overall survival, the AUC values were 0.558 (0.491–0.626), 0.579 (0.540–0.618), and 0.562 (0.527–0.597) at 1, 3, and 5 years, respectively.

## 4 Discussion

The etiology of NPC is unique and complex, and it remains incompletely understood. Although the incidence of NPC is low in most parts of the world, it is endemic in a few well-defined populations, including natives of Southern China, Southeast Asia, the Arctic, and the Middle East/North Africa (43, 44). Previous evidence suggests that NPC is associated with multiple risk factors, including EBV infection (45), genetic susceptibility (46), and environmental factors (47). Given that NPC shows a high sensitivity to radiotherapy, combined chemoradiotherapy, which incorporates induction chemotherapy, is frequently employed for comprehensive management (6). Despite an overall 5-year survival rate of approximately 80% in patients with NPC, 20–30% of them develop distant metastasis and/or local recurrence, with bone being the most common site of metastasis (10, 14). Once metastasis is diagnosed, the average OS for these patients usually falls below 15 months with palliative chemotherapy (14). Therefore, identifying independent prognostic factors for NPC, particularly easily accessible biomarkers to predict outcomes or prognosis, can help in developing individualized treatment regimens, thereby improving outcomes for patients with NPC (33). To our knowledge, this is the first study to establish pre-treatment AMC as an independent predictor of metastatic progression and mortality in treatment-naive non-metastatic NPC. Our findings suggest that AMC’s prognostic utility manifests through several interconnected dimensions, including its capacity for threshold-dependent risk stratification, distinct non-linear relationships with clinical endpoints, consistency across patient subgroups, and clinically relevant diagnostic performance. Collectively, these insights significantly advance risk stratification paradigms beyond conventional TNM staging, addressing the urgent need for biomarkers that capture tumor biological heterogeneity in NPC management.

The optimal AMC cut-off value of 0.63×10^9^/L, derived through maximally selected rank statistics, helps in effectively delineating populations with divergent metastatic trajectories. The results showed that patients with high AMC had worse DMFS, BMFS, and OS. Notably, patients with high AMC (≥0.63×10^9^/L) exhibited a 1.86-fold increased risk of bone metastasis in fully adjusted models—the strongest association observed among all endpoints. The specificity for skeletal dissemination aligns with emerging evidence of monocyte-mediated osteolytic activation. Monocytes infiltrating the tumor microenvironment differentiate into osteoclastogenic macrophages that secrete receptor activator of nuclear factor-κ B ligand (RANKL), interleukin (IL)-6, and matrix metalloprotein (MMP)-9—enzymes critical for bone matrix degradation and metastatic niche formation (48). The preferential homing of monocyte-derived cells to bone marrow may explain why a disproportionate increase in AMC aided in better prediction of skeletal involvement than general distant metastasis (HR = 1.33). RCS analysis uncovered fundamental differences in AMC’s relationship with clinical outcomes. While distant metastasis (P for non-linearity=0.630) and bone metastasis (P for non-linearity=0.081) demonstrated linear dose-response relationships with increasing AMC, overall mortality rates exhibited a distinct threshold effect (P for non-linearity=0.011) with the risk increased with AMC >0.54×10^9^/L, eventually reaching a plateau. This divergence suggests that while monocyte abundance continuously fuels metastatic progression through angiogenesis and immunosuppression, its impact on survival may become saturated once a critical immunosuppressive threshold is crossed (48–50). Alternatively, the mortality plateau may reflect competing risks from non-metastatic causes in advanced disease—a hypothesis warranting investigation in prospective studies. The consistency of AMC’s prognostic value across all pre-specified subgroups (P for interaction >0.05) is an important observation. Crucially, AMC maintained predictive power regardless of treatment modality; this treatment-agnostic effect underscores that AMC captures fundamental tumor–host biology rather than treatment responsiveness. Such consistency across heterogeneous patient strata adds weight to the consideration of incorporating AMC into existing prognostic frameworks. However, the exact underlying mechanisms of the association between increased AMC and poor outcomes remain unclear and may involve multiple factors (50).

Inflammation plays a significant role in cancer progression, and chronic systemic inflammatory responses are strongly associated with the progression process and subsequent poor outcomes in patients with cancer (51). Tumor cells interact with non-cancerous cells in the extracellular matrix, such as inflammatory and immune cells, secreting inflammatory factors to form a unique tumor inflammatory microenvironment, which significantly influences tumor malignancy (20). Inflammatory cells in the blood include neutrophils, lymphocytes, monocytes and other cell types. AMC is independently associated with the prognosis of various tumors, such as non-small cell lung cancer, head and neck squamous cell carcinoma, prostate cancer, and metastatic NPC (52–55). Immune and inflammatory responses in the microenvironment play a key role in tumor initiation, progression, and metastasis. The prognostic and predictive value of circulating inflammatory/immune cell counts has been reported in many cancer types. A characteristic feature of NPC is the infiltration of leukocyte subtypes, such as lymphocytes, monocytes, and neutrophils (56). Monocytes and their derived cells, such as myeloid-derived suppressor cells and tumor-associated macrophages (TAMs), are frequently found in the TME and play a critical role in inflammation and cancer (38). Tumor-derived factors attract monocytes from peripheral blood into tumor tissue, where they differentiate into TAMs, a key component of inflammatory infiltration (57). High TAM density in the tumor stroma is associated with poor prognosis in NPC similar to that in other tumor types (58). Monocytes can secrete various pro-inflammatory cytokines, such as interleukin (IL)-1, IL-6, IL-10, and tumor necrosis factor-α (TNF-α), which have been associated with shortened survival and poor prognosis in patients with malignant tumor (59, 60). Additionally, monocytes can release monocyte chemo-attractant protein (MCP)-1 upon stimulation and mediate tumor-associated macrophage infiltration in solid tumors, which can produce various chemokines such as transforming growth factor-α (TGF-α), TNF-α, IL-1, and IL-6, to promote tumorigenesis, angiogenesis, and distant metastasis in malignant tumors (61, 62).

In summary, peripheral monocyte counts are closely associated with TAMs, which differentiate within the TME and secrete various cytokines and growth factors to induce tumor progression (63). These cytokines and growth factors can induce angiogenesis and anti-immune effects. Therefore, a high AMC may indicate a poor prognosis. In this study, we evaluated AMC as a prognostic indicator in 2,046 patients with newly diagnosed treatment-naive, non-metastatic NPC. Some of our findings are consistent with those of previous reports (42, 54). Our multivariate Cox proportional hazards models indicate that elevated pre-treatment AMC is independently associated with metastatic progression and diminished survival outcomes in patients with treatment-naive NPC, with particularly strong associations observed for bone metastasis (HR = 1.86, 95%CI 1.33–2.60). However, some of our results differ from those reported by He et al. (64), who showed that the percentage of peripheral blood monocytes was not associated with OS and DFS in NPC. In the current study, pre-treatment peripheral AMC remained an independent prognostic factor for OS after adjusting for confounding factors. The discordance between these two studies may be partially due to difference in follow-up duration, where the median duration was 77.4 months in the present study and 41 months in the previous study. Nevertheless, Lin et al. (65) reported that peripheral AMC was not associated with OS in patients with metastatic NPC undergoing chemotherapy, and AMC had no prognostic significance in COX multivariate analysis. The inconsistency between the two studies may be partially attributed to different sample sizes, as 2,046 patients were recruited in the present study compared with 256 in Lin et al.’s study. In the present study, elevated pre-treatment peripheral AMC (≥ 0.63×10^9^/L) were closely associated with worse OS in NPC under univariate and multivariate analyses. This finding is consistent with Jiang et al.’s findings (54), indicating that reduced monocyte count (<0.665×10^9^/L) was significantly associated with prolonged OS in patients with NPC. A monocyte count ≥0.665 × 10^9^/L is an independent prognostic indicator of unfavorable prognosis of survival endpoints in patients with metastatic NPC (HR = 1.98, 95%CI = 1.63–2.41, P < 0.001). Additionally, Li et al. (42) found that the 5-year OS HR of patients with monocyte count ≥0.475×10^9^/L compared to <0.475×10^9^/L was 1.409 (95%CI: 1.078–1.843). Lower monocyte count (<0.475×10^9^/L) indicated better OS (P = 0.012), DFS (P = 0.011), and DMFS (P = 0.003) in NPC. Although an increase in monocyte count may be an unfavorable prognostic factor for cancer prognosis, the underlying mechanisms between monocytes and cancer progression remain unclear.

We applied the maximally selected rank statistics method to determine the optimal AMC cut-off value at 0.63×10^9^/L, which is comparable to the cut-off value reported by Jiang et al.’s findings, but differs from that reported by Li et al.’s findings. This consistency with prior findings supports the feasibility and effectiveness of using the maximally selected rank statistics method for determining the AMC cut-off value in this study. The observed discrepancies may stem from methodological variations in cut-off value determination. Moreover, differences in population characteristics—particularly between non-metastatic and metastatic cohorts—may further contribute to the variation in optimal cut-off value. In our opinion, this AMC cut-off value may serve as a complementary biomarker to assist in predicting survival and tumor metastasis in patients with NPC. However, due to the absence of standardized decision cut-off value and the limited specificity of AMC, the derived cut-off value cannot be used independently or universally for clinical diagnosis of specific conditions. Clinical interpretation should integrate other laboratory parameters—such as complete blood count test and imaging findings to enable more accurate health assessment and disease diagnosis. Future multicenter studies employing standardized methodologies are required to establish clinically applicable AMC cut-off value.

Furthermore, our time-dependent ROC analysis provides a nuanced view of AMC’s prognostic utility across the disease timeline. The notably high 1-year AUC for bone metastasis (0.720) underscores its potential role in early risk stratification, which could be critical for selecting patients who might benefit from more intensive surveillance or early intervention. The sustained, albeit moderate, AUC values for overall survival across 3 and 5 years reinforce that pre-treatment AMC captures a persistent risk signal beyond initial treatment phases. This dynamic profiling aligns with the biological role of monocytes in fostering a pro-metastatic microenvironment and supports the integration of AMC into time-sensitive prognostic models for NPC.

Numerous studies have confirmed the potential role of blood inflammatory markers in cancer prognosis, but their limitations should not be overlooked. These blood inflammatory markers do not exhibit a uniform cut-off value. This variability likely arises from multiple factors, with methodological divergences in cut-off value determination representing a key contributor. Specifically, our application of maximally selected rank statistics differs substantially from ROC curve methods used in referenced studies (42, 54, 65). While ROC curves provide intuitive classification accuracy metrics, they are limited in handling censored time-to-event data (66). In contrast, maximally selected rank statistics are designed for continuous or ordinal responses that may be censored, such as survival endpoints, incorporating censoring information through rank-based weighting using log-rank scores. This approach handles tied observations using mid-scores and ensures valid inference through conditional variance estimation, making it suitable for analyzing prognostic factors with censored outcomes (67). Consequently, while population heterogeneity, particularly between non-metastatic and metastatic cohorts, undoubtedly influences threshold variation, methodological differences offer a compelling explanation for the disparity between our maxstat-derived cut-off value (0.63×10^9^/L) and Li et al.’s ROC-based threshold of 0.475×10^9^/L (42). These findings highlight how analytical approaches interact with clinical variables to shape the interpretation of prognostic biomarkers. Finally, certain underlying conditions, such as anemia, chronic inflammation, or impaired liver and kidney function, may influence the levels of corresponding serum markers emphasizing the need for set standardized thresholds for blood inflammatory markers (33).

AMC can be easily obtained from a simple complete blood count test, and its application in routine clinical practice is technically and economically feasible. Although we found an association between inflammatory biomarkers and prognosis, this study has some limitations. Firstly, this was a retrospective study, and data were obtained from a single center. Therefore, we were unable to control for potential positive or negative biases during treatment or patient selection, warranting external validation in future multicenter prospective studies.

Secondly, the absence of longitudinal AMC measurements during treatment. Compared with traditional baseline values, dynamic monitoring of AMC changes during treatment may more accurately reflect alterations in the tumor microenvironment and the impact of therapy on the patient’s immune status, thereby enabling improved prediction of disease progression, recurrence risk, and treatment response. Although our study primarily focused on pre-treatment AMC, emerging evidence indicates that longitudinal assessment of inflammatory markers throughout therapy may yield greater prognostic insight. Tracking AMC trends throughout the therapeutic course may facilitate early identification of non-responders or patients experiencing disease progression. Such an approach can offer real-time clinical guidance for treatment modification, thereby enhancing the translational relevance and clinical applicability of research findings (56, 68). Additionally, although our model was adjusted for key clinical variables—including age, sex, TNM stage, and treatment modalities—data on several important prognostic factors were unavailable, such as EBV–DNA load, performance status, comorbidities, and socioeconomic factors. Of particular note, EBV–DNA is a well-established prognostic biomarker in NPC, and its omission represents a significant limitation. The absence of these variables may compromise the accuracy and reliability of our findings regarding NPC prognosis and could diminish the predictive performance of the model. Therefore, future studies should incorporate these covariates to confirm the independent prognostic value of AMC, and investigate whether serial measurements of AMC during therapy confer incremental prognostic value over baseline assessments alone.

Thirdly, AMC is notably influenced by physiological states and exhibits substantial inter-individual variability, resulting in a wide normal range and a lack of standardized decision cut-off value (69). Additionally, AMC lacks disease specificity, as alterations may occur in various conditions, including infections, chronic inflammatory diseases, comorbidities, autoimmune disorders, and hematologic diseases (70–75). Although pre-treatment AMC demonstrated significant and independent prognostic value, its time-dependent discriminatory accuracy (AUCs ranging from 0.555 to 0.720) is moderate. This suggests that its clinical utility for risk stratification may be maximized when integrated with other established factors. Consequently, future studies should prioritize exploring its interactions and synergistic effects with other biomarkers within composite models. Understanding these collective impacts will be crucial for developing more precise prognostic strategies and enhancing clinical decision-making.

Fourthly, given the extended duration of this study (2009–2022), changes in treatment standards, updates to diagnostic and therapeutic guidelines, and the introduction and widespread use of novel therapies or drugs during this period represent important confounding factors in long-term survival analyses. These variables may directly influence the interpretation of results and compromise intergroup comparability. Although we adjusted for broad categories of treatment modalities (e.g., radiotherapy, chemotherapy), detailed information on specific regimens, dosages, and administration protocols was not available. Such variability in treatment approaches may constitute a potential confounding factor that independently affects patient survival outcomes beyond tumor marker levels. Therefore, future studies should incorporate time-dependent covariate adjustments to enhance the accuracy of survival analysis.

Finally, although key demographic characteristics were balanced between included and excluded patients, residual selection bias from unmeasured factors cannot be excluded. Furthermore, the absence of prospective screening for acute infections at baseline may have led to confounding by unmeasured subclinical inflammation. Although sensitivity analyses supported the robustness of our findings, future multicenter prospective studies with systematic inflammatory assessment are needed.

Overall, our findings indicate that pre-treatment peripheral AMC levels are associated with the prognosis of NPC, and may be considered as an independent risk factor. In conclusion, our analysis revealed that AMC ≥0.63×10^9^/L is significantly associated with poorer DMFS, BMFS, and OS in NPC than AMC <0.63×10^9^/L. Therefore, an AMC ≥0.63×10^9^/L can be considered as an adverse prognostic factor for DMFS, BMFS, and OS in patients with newly diagnosed treatment-naive, non-metastatic NPC.

## 5 Conclusion

Our findings suggest that pre-treatment peripheral AMC may serve as an independent prognostic factor for patients with newly diagnosed treatment-naive, non-metastatic NPC. The detection of AMC can be easily determined from complete blood count and is readily applicable clinically, making it a practical complementary prognostic indicator of NPC. Further external validation in prospective, multicenter cohorts is warranted to confirm these findings and establish the clinical utility of AMC in NPC prognosis.

## Data Availability

The original contributions presented in the study are included in the article/**Supplementary Material**. Further inquiries can be directed to the corresponding authors.
