# Supplementary material for: Pre-treatment peripheral absolute monocyte count predicts metastatic progression and survival outcomes in treatment-naive non-metastatic nasopharyngeal carcinoma
Source: Front Oncol. 2026 Feb 17;16:1696050. doi: 10.3389/fonc.2026.1696050 (PMC12953105; doi:10.3389/fonc.2026.1696050)
Supplement: Supplementary file 1 [file Table1.docx]

**Supplementary Tables**

**TABLE S1** General characteristics for patients between included and excluded groups.

| **Variables** | **Included**  **(n=2046)** | **Excluded**  **(n=1503)** | **P value** |
| --- | --- | --- | --- |
| Age (years) | 51.00(44.00,59.00) | 51.00(43.00,59.00) | 0.333 |
| Male, n(%) | 1515.00(74.05) | 1091.00(72.59) | 0.350 |
| TNM stage, n(%) |  |  | 1.000 |
| Ⅰ-Ⅱ | 214.00(10.46) | 158.00(10.51) |  |
| Ⅲ-Ⅳ | 1832.00(89.54) | 1345.00(89.49) |  |
