# Supplementary material for: Pre-treatment peripheral absolute monocyte count predicts metastatic progression and survival outcomes in treatment-naive non-metastatic nasopharyngeal carcinoma
Source: Front Oncol. 2026 Feb 17;16:1696050. doi: 10.3389/fonc.2026.1696050 (PMC12953105; doi:10.3389/fonc.2026.1696050)
Supplement: Supplementary file 2 [file Table2.docx]

**Supplementary Tables**

**Table S2.** Sensitivity analyses of the association between high pre-treatment AMC (≥0.63×10⁹/L) and survival outcomes in fully adjusted models.

| **Analysis Cohort** | **N after exclusion** | **Endpoint** | **HR(95%CI)** | **P-value** |
| --- | --- | --- | --- | --- |
| Primary Analysis | 2046 | DMFS | 1.33 (1.05-1.70) | 0.020 |
|  |  | BMFS | 1.86 (1.33-2.60) | <0.001 |
|  |  | OS | 1.34 (1.11-1.61) | 0.002 |
| Sensitivity Analysis 1 | 2024 | DMFS | 1.31 (1.03-1.67) | 0.029 |
|  |  | BMFS | 1.83 (1.30-2.57) | <0.001 |
|  |  | OS | 1.34 (1.12-1.62) | 0.002 |
| Sensitivity Analysis 2 | 1732 | DMFS | 1.39 (1.06-1.82) | 0.017 |
|  |  | BMFS | 2.00 (1.38-2.92) | <0.001 |
|  |  | OS | 1.36 (1.11-1.67) | 0.003 |

All models are adjusted for age, sex, TNM stage, radiotherapy, chemotherapy.

Sensitivity Analysis 1: Excludes patients with AMC values ≤0.5th percentile and ≥99.5th percentile of the cohort distribution (N excluded=20).

Sensitivity Analysis 2: Excludes patients seropositive for HBsAg and anti-HCV (N excluded=314).
